# Effects of soil fertility and toxicity on the performance of *Chenopodium quinoa* (Willd) plants in kin and non-kin interactions

**DOI:** 10.1080/15592324.2025.2591495

**Published:** 2025-12-01

**Authors:** Jan Sher, Yun-Bing Zhang, Jiao-Lin Zhang

**Affiliations:** a Center for Integrative Conservation & Yunnan Key Laboratory for Conservation of Tropical Rainforests and Asian Elephants, Xishuangbanna Tropical Botanical Garden, Mengla, People's Republic of China; b Yunnan Key Laboratory of Forest Ecosystem Stability and Global Change, Xishuangbanna Tropical Botanical Garden, Chinese Academy of Sciences, Mengla, People's Republic of China; c University of Chinese Academy of Sciences, Beijing, People's Republic of China

**Keywords:** Biomass allocation, *Chenopodium quinoa*, competition, fertility, Kin selection, Niche partitioning, toxicity

## Abstract

Kin selection theory predicts that closely related organisms may exhibit cooperative behaviors that enhance group fitness despite individual costs. In contrast, the resource partitioning hypothesis posits stronger competition among close relatives due to shared resources and niche overlap. In this study, we tested whether quinoa (*Chenopodium quinoa* Willd) genotypes differ in performance when grown with kin versus non-kin under different soil fertility and heavy metal toxicity conditions. A two-level, three-factorial experimental design was conducted, including kinship, fertility, and toxicity. Biomass accumulation, allocation patterns, resource acquisition traits, and photosynthetic parameters were measured at the end of the experiment. Kinship and fertility effects were common, but toxicity effects were rare. Biomass accumulation was greater in more fertile soils, and kinship marginally increased biomass. Root allocation was affected by toxicity interactions: kin plants showed greater root allocation under no-toxicity conditions, but this difference was suppressed under metal toxicity. Resource acquisition traits reflected these patterns, with specific taproot length and average leaf mass being higher for kin combinations, while specific stem length and specific leaf area were higher for non-kin combinations. The net assimilation rate, stomatal conductance, transpiration rate, and WUEi were generally higher in non-kin than in kin plants, regardless of soil fertility. These results suggest that quinoa plants may benefit from kin interactions through increased root growth and overall biomass accumulation, but metal toxicity suppresses these benefits, showing that kinship advantages are context dependent and reduced in contaminated soils.

## 1 Introduction

Kin recognition in organisms represents a critical social behavior that can drive the evolution of altruism.^1-3^ The theory of kin selection^4^ proposes that cooperation and competition among individuals depend upon their genetic relatedness, favoring behaviors that enhance the reproductive success of their relatives even at personal fitness costs. Plants achieve kin recognition through root and shoot signaling,^1^^,^^5^^,^^6^ which may mitigate competitive reductions in growth and survival.^7^^,^^8^ Under resource limitation, such recognition potentially reduces costly competition while promoting cooperative advantages among kin.^9^^,^^10^ In contrast to the expectations of kin selection theory, the niche partitioning ecological hypothesis predicts that competition between close relatives will likely be stronger because they compete for more similar resource niches,^9-14^ meaning that kin interactions will be more deleterious than non-kin interactions. Empirical evidence remains contradictory: some studies report superior performance in kin groups,^5^^,^^10^^,^^15-19^ other studies reported reduced performance,^20-22^ while several detected no significant differences.^22-24^ This inconsistency underscores the need to resolve the contextual drivers of these divergent patterns. Cheplick and Kane^25^ used *Triplasis purpurea* to investigate the effects of genetic relatedness on competition among plants, specifically focusing on whether kin selection or resource partitioning mechanisms are at play. They observed that kin plants were able to partition resources more effectively than non-kin plants suggesting that genetic relatedness may facilitate cooperative resource sharing. Our study extends this investigation using quinoa (*Chenopodium quinoa* Willd.) to disentangle these mechanisms under variable abiotic stresses.

It remains unclear how soil properties that induce plant stress, such as nutrient limitation and toxicity, alter the benefits of kin selection. Nutrient levels may alter the costs of cooperation versus competition.^13^^,^^26^ Plants in low-nutrient environments may allocate a greater proportion of resources to roots to enhance their uptake of limiting soil nutrients,^27^ for example, by increasing their specific root length and lateral root production in response to soil nutrient limitation.^28^^,^^29^ Importantly, plants integrate cues from both neighbor identity and nutrient availability to optimize competitive strategies.^26^ Detection of belowground neighbors can trigger root growth before resources run out,^30^ suggesting a proactive response to the presence of neighboring roots rather than just a reaction to limited resources.^31^

Soil heavy metal pollution creates toxic soil conditions, and is a prominent environmental problem associated with industrialization.^32^^,^^33^ These toxicants can alter plant structure and functional properties, inhibit seed germination,^33^^,^^34^ inhibit root elongation and lower mineral uptake, and reduce photosynthesis in plants, leading to reductions in crop growth and quality.^32^ Heavy metals induce nutritional deficiencies, suppress chlorophyll biosynthesis, reduce photosynthesis, generate oxidative stress, and in severe cases, cause plant mortality.^35^ Whether toxicity damages plant signaling is unclear, but if it does so, then toxicity may inhibit the signaling that promotes kin-to-kin mutualistic behavior. Plant responses to toxicity are regulated by the differential expression of genes, the enhancement of antioxidant-system activity and by the synergistic crosstalk between signal molecules.^35^^,^^36^ However, it is unclear how soil fertility and toxicity interact to alter the benefits/costs of kin versus non-kin plants.

*Chenopodium quinoa* Willd. (Amaranthaceae), commonly known as quinoa, has gained attention globally due to its high nutritional content.^10^^,^^37^ Quinoa is native to the Andean region of South America and is now cultivated globally, including in Bolivia, Peru, United States, Ecuador and Canada.^38-40^ Quinoa is resistant to multiple abiotic stresses (e.g. drought, salinity, and frost) relative to other crops.^41^ Notably, quinoa accumulates substantial heavy metals in leaf tissue during early growth stages^37^ though excessive metal concentrations ultimately reduce biomass production.^42^^,^^43^ Despite these well-characterized stress responses, the interactive effects of soil fertility and heavy metal toxicity on kin-mediated growth strategies in quinoa remain unexplored. Resolving this knowledge gap could inform optimal cultivation practices by determining whether kin associations enhance productivity under varying soil conditions.

In this study, we tested how quinoa growth performance, biomass allocation patterns, and photosynthesis respond to kin versus non-kin interactions under contrasting soil fertility and heavy metal toxicity conditions. Understanding the responses of crops to adverse growing conditions is important to develop agricultural adaptation strategies.^38^ To our knowledge, no previous study has examined the combined effects of soil fertility and toxicity stress on quinoa performance in the context of kin versus non-kin combinations. We used two different levels of nutrient (poor and rich) to represent nutrient availability, two levels of soil toxicity (metal versus non-metal), and four different genotypes of quinoa to represent kin combinations (same genotype) and non-kin combinations (different genotypes). In this study, we addressed the following questions: (1) How do kin versus non-kin associations influence quinoa growth, morphology and physiology? (2) How do soil fertility and toxicity affect the growth, morphology and physiology of quinoa when grown with kin and non-kin genotypes. We hypothesize that kin plants will compete more intensely for resources than non-kin plants because genetically similar individuals are expected to use more similar resource niches (niche partitioning hypothesis)(H1). (a) Under poor soil conditions, this niche overlap will increase belowground competition among kin, leading to greater investment in root traits (e.g. higher root allocation, longer taproots), which may reduce aboveground biomass. (b) Under rich soil conditions, competition is expected to be weaker, and differences in resource allocation and growth between kin and non-kin plants should be smaller. We also hypothesized that toxic soil conditions would suppress plant growth and reduce investment in both belowground and aboveground traits. Under strong toxicity, overall biomass is expected to decrease, and differences between kin and non-kin combinations should become small because toxicity limits plants ability to compete, regardless of genetic relatedness (H2).

## 2 Materials and methods

### 2.1 Seed collection and germination in greenhouse

Seeds of *Chenopodium quinoa*, all from the highland ecotype, were obtained from the quinoa gene bank at the Universidad Nacional del Altiplano in Peru. Four genotypes (B2, BR2, Y2, and W23; Table 1) were germinated in a greenhouse at the Xishuangbanna Tropical Botanical Garden, Chinese Academy of Sciences, Mengla County, Yunnan Province, China. The seeds were germinated on 20 January 2020. The daytime temperature ranged from 22 to 26 °C, and the nighttime temperature ranged from 18 to 24 °C. River sand was used as the growth medium for seed germination. After eight days, healthy seedlings of similar size were selected and transplanted into pots.^10^^,^^18^

**Table 1.** Origin and morphological characters of the quinoa cultivars used in the experiment, adapted from Cai and Gao (2020).^44^

Genotype	Local code	Origin	Photoperiod sensitivity	Other characters
#35	B2	Peru (Puno)	Neutral day	White stem and inflorescence, green leaves, less tolerant to frost. Late maturing. Brown seeds; seed weight: 0.00297 g per seed
#03	R1	Peru (Puno)	Short-neutral day	Red stem and inflorescence, red young leaves, tolerant to frost and downy mildew. Early maturing. black seeds; seed weight: 0.00272 g per seed
#14	Y2	Bolivia (southern altiplano)	Short day	Yellow stem. Panicles are colored from white to yellow. Late maturing. Yellow seeds; seed weight: 0.00486 g per seed
#45	W23	W23	Short day	White stem and inflorescence; tolerant to frost and drought. Early maturing. Red seeds; seed weight: 0.00292 g per seed.

### 2.2 Treatments

We conducted a two-level three-factorial pot experiments, including neighbor kinship (kin versus non-kin), soil fertility (poor versus rich soil), and toxicity (heavy metal versus non-heavy metal). Seedlings were selected from seeds from four mother plants in each genotype. Seeds that were collected from the same genotype were considered as kin; while seeds collected from different genotypes were considered as non-kin^10^^,^^18^ The size of each pot was 44 cm long and 28 cm wide and 10 cm deep. Kin pots contained seedlings from a single genotype, whereas non-kin pots contained seedlings from different genotypes. In non-kin pots, seedlings of two different genotypes were interspersed. The genotypic identity of each seedling was known, allowing us to account for differences in growth potential between seedlings due to genotype (a random effect). Six seedlings were planted 6 cm apart in each pot.^10^^,^^18^

For the soil fertility treatment, we added 20 g kg^−1^ NPK 20:20:20 into the soil medium for the preparation of rich soil prior to the start of the experiment, while for the poor soil we did not add any nutrients. All pots contained 10 kg soil. For the heavy metal toxicity treatment, we mixed 100 mg kg^−1^ lead nitrate (Pb (NO_3_)^2^) into the soil medium as a heavy metal toxicity treatment and no lead nitrate was added into the no heavy metal toxicity treatment. The heavy metal was added to the pots 10 days after the seedlings had been transferred into the pots.

In total, there were 80 pots: 40 pots for toxicity treatment (20 pots with poor soil and 20 pots with rich soil) and 40 pots for the no-toxicity treatment (20 pots with poor soil and 20 pots with rich soil). Each pot contained 6 plants, including four kinship combinations and six non-kinship combinations, totaling 480 seedlings. All pots were kept well-watered by adding 500 ml water twice in a week, to maintain them close to water holding capacity.

### 2.3 Morphological traits and biomass measurement

The plants were harvested 80 d after planting, prior to seed production, to avoid heat damage from the rising greenhouse temperatures in spring.^10^ The following measurements were taken on each individual plant: total root length (cm, the sum of lengths of all individual roots), stem length (cm, the length of the primary axis), dry mass (g, dried in an oven at 65 °C for 48 h) of roots, stems, and leaves. Dry mass data were used to calculate total dry mass (TDM, g), shoot dry mass (SDM, g), which served as proxies for plant performance (TDM for total growth, SDM for aboveground growth).^10^^,^^18^ Plant biomass is considered to be a reliable proxy for fitness when seed production is not measured.^10^ We used organ mass fractions, leaf mass fraction (LMF, g leaf g^−1^ total mass), stem mass fraction (SMF, g stem g^−1^ total mass), and root mass fraction (RMF, g root g^−1^ total mass), to understand biomass partitioning. We also measured resource acquisition traits, specific taproot length (STRL, m g^−1^), specific stem length (SSL, m g^−1^), average leaf mass (ALM, g), and specific leaf area (SLA) as since larger values indicate greater resource uptake efficiency (soil nutrients, soil water, light) per unit biomass.^10^ All measurements were conducted at the individual plant level.

### 2.4 Photosynthetic rate measurement

To measure the photosynthetic rate of all quinoa varieties, gas exchange, stomatal conductance, and vapor pressure deficit were recorded using a LI−6800 system (Li-COR Inc., Lincoln, Nebraska, USA). Measurements were taken 60 d after planting (about 20 d before harvest) on six leaves per plant, selected from different parts of the canopy. Measurements were conducted once for each plant. The CO₂ concentration, relative humidity, and leaf temperature inside the cuvette were maintained at 400 µmol mol⁻ ¹ , 65%, and 28 °C, respectively. Based on the light response curve, the photosynthetic photon flux density (PPFD) in the cuvette was set to 1500 µmol m⁻² s⁻ ¹ . Leaves were placed in the cuvette, and readings were taken once the photosynthetic rate stabilized (usually within 30 s). Data were recorded when the change in photosynthetic rate was less than 0.5% over one minute. Outdoor *in situ* gas exchange estimation was conducted between 8 am and 10 am using the standard methods.^45^^,^^46^ The environmental conditions in the cuvette were set as described above, and the system was allowed 15–20 min to stabilize before recording measurements, ensuring that changes in photosynthetic rate were less than 0.5% within one minute.

### 2.5 Statistical analysis

All statistical analyzes were conducted using R 4.0.3. We used a linear mixed effects model to analyze the data, using the ‘lmer’ function of the *lme4* package with kinship, fertility and toxicity treated as fixed effects in a full interaction model with genotype identity and pot identity included as a random effects (random intercepts only).^10^ We tested these effects on all response variables, namely total dry mass, shoot dry mass, organ mass fractions (LMF, SMF, RMF), resource acquisition traits (STRL, SSL, ALM, and SLA), and photosynthetic parameters (assimilation rate, transpiration rate, stomatal conductance and WUEi). These response variables were transformed as necessary to achieve approximate normality prior to regression analysis. Coefficient significance was evaluated using t tests with Satterthwaite approximation of error degrees of freedom. Explanatory power (estimated R^2^ values) was estimated using the ‘r.squaredGLMM’ function of the MuMIn package.^47^^,^^48^ Subsequently, pairwise Tukey HSD tests were conducted on the groups in the chosen model using the ‘glht’ function of the *multcomp* package.^49^

## 3 Results

### 3.1 Biomass accumulation and allocation

**Figure 1.**
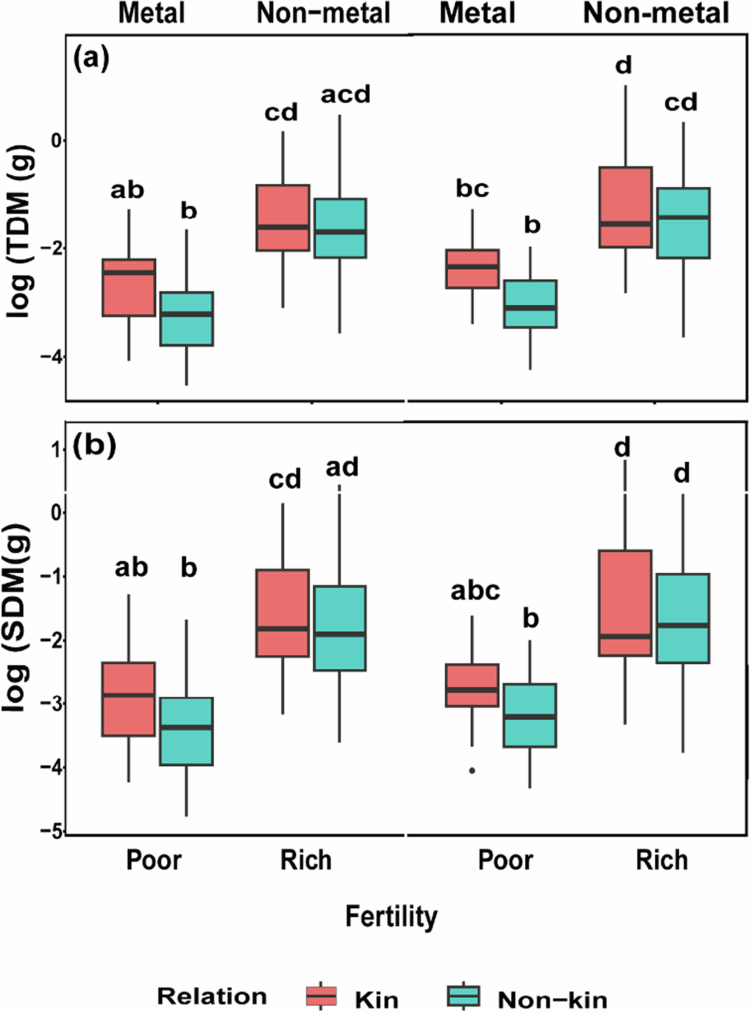
Biomass accumulation in all treatment combinations. (a) Total dry mass (TDM) and (b) shoot dry mass (SDM) of kin and non-kin in all treatment combinations, including poor versus rich soil and metal, versus non-metal treatments. Common letters within each panel indicate no statistically significant difference among the treatment pairs in that panel (based on Tukey HSD tests on transformed data).

Total dry mass (TDM) and shoot dry mass (SDM) per pot differed significantly across kinship, fertility, and toxicity treatments (Table 2, Figure 1), being greater with increased fertility, and differed marginally with kinship, being greater for kin combinations than non-kin combinations.

**Table 2.** Summarized statistical results for biomass accumulation, biomass allocation and resource acquisition traits variables in the full model. Transformations applied to normalize response data prior to regression are indicated. Random effect standard deviances are given. Alternative case levels for fixed effects are indicated for coefficients. Coefficients are evaluated using t tests based on Satterthwaite approximation of error degrees of freedom.

	TDM (log)	SDM (log)	SMF	LMF (log)	RMF (log)	STRL (log)	SSL (log)	ALM (log)	SLA (log)
Random effects (variance)									
PotID	0.33	0.35	0.10	0.10	0.39	0.59	0.50	0.64	0.76
Genotype	0.02	0.01	0.02	0.02	0.04	0.00	0.16	0.00	0.00
Residual	0.43	0.49	0.16	0.15	0.82	0.93	0.75	0.68	0.77
Fixed effects									
Intercept	**−2.752**	**−2.997**	**0.592**	**2.767**	**−2.196**	**2.219**	**1.651**	**−6.187**	**5.309**
Total dry mass		**1.476**	**0.059**	7.584	**−0.263**	**−1.096**	**−1.480**	**1.122**	**−0.401**
Relation: non-kin	**−0.696**	**−0.489**	−0.004	6.828	**−0.179**	0.159	**0.580**	−0.109	**−0.084**
Fertility: rich	**0.880**	**0.874**	−0.095	7.777	0.266	**−0.648**	**−0.308**	**0.943**	**−0.109**
Toxicity: non-metal	0.209	0.0686	**−0.163**	6.433	**0.660**	**−0.504**	**0.773**	0.250	**−0.381**
Relation: non-kin × fertility: rich	0.355	0.332	−0.015	1.463	−0.249	−0.327	−0.153	0.137	1.221
Relation: non-kin × toxicity: non-metal	0.027	0.170	0.113	−1.298	−0.626	0.213	−0.448	−0.749	0.296
Fertility: rich × toxicity: non-metal	−0.256	−0.232	0.070	−3.854	−0.437	0.178	−0.230	−0.172	0.664
Relation: non-kin × fertility: rich × Toxicity:non-metal	0.228	0.146	0.004	−7.869	0.659	−0.070	−0.184	0.016	−0.471
**R** ^**2**^ **m**	0.73	0.70	0.08	0.05	0.08	0.40	0.52	0.50	0.20
**R** ^**2**^ **c**	0.83	0.80	0.41	0.35	0.36	0.49	0.67	0.74	0.60

Note: Bold values indicate coefficients significant at *p* < 0.05 are emboldened.

**Figure 2.**
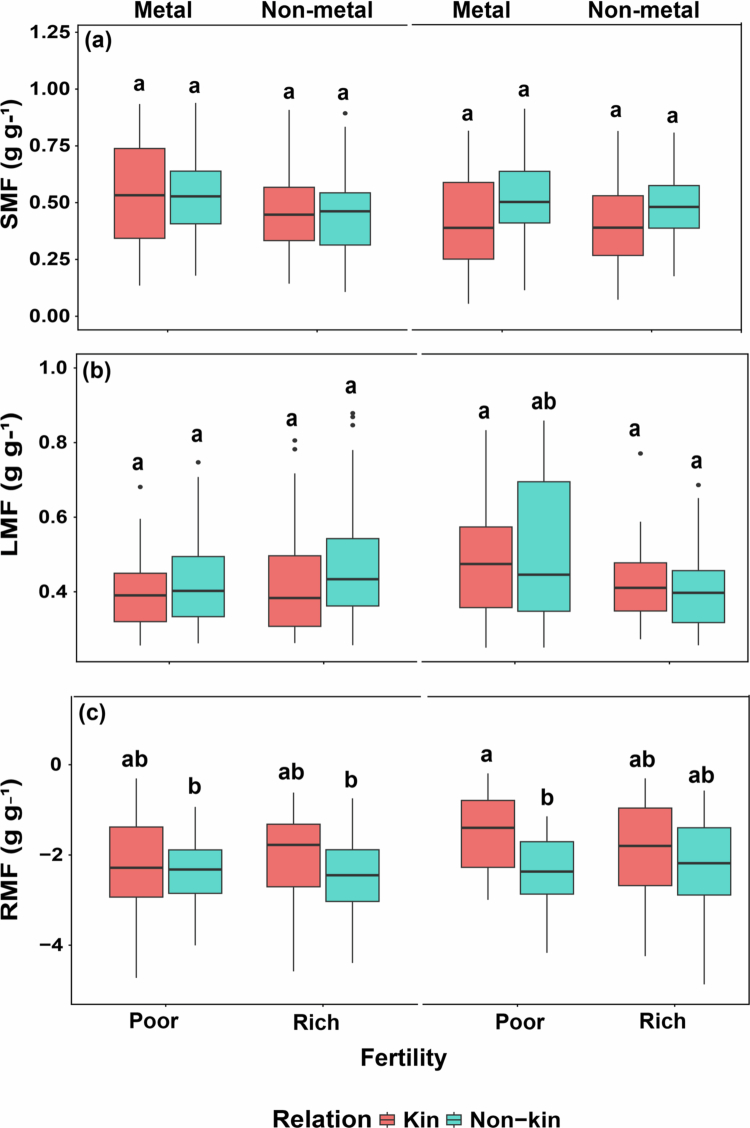
Biomass allocation in all treatment combinations. (a) Stem mass fraction (SMF), (b) leaf mass fraction (LMF), and (c) root mass fraction (RMF) of kin and non-kin under poor versus rich soil fertility and metal versus non-metal treatments. Common letters within each panel indicate no statistically significant difference among the treatment pairs in that panel (based on Tukey HSD tests on transformed data).

Stem mass fraction (SMF) was impacted only by toxicity (Table 2, Figure 2a), being significantly lower in kin plants than non-kin plants. Leaf mass fraction (LMF) was not impacted by any of the predictors.

Root mass fraction (RMF) was significantly influenced by fertility and toxicity (Table 2, Figure 2c). Kin plants had greater RMF than non-kin plants under soil fertility and toxicity, but metal toxicity suppressed RMF and significantly more so for kin, such that there was less difference in RMF of kin versus non-kin in the high metal treatment.

### 3.2 Resource acquisition traits

**Figure 3.**
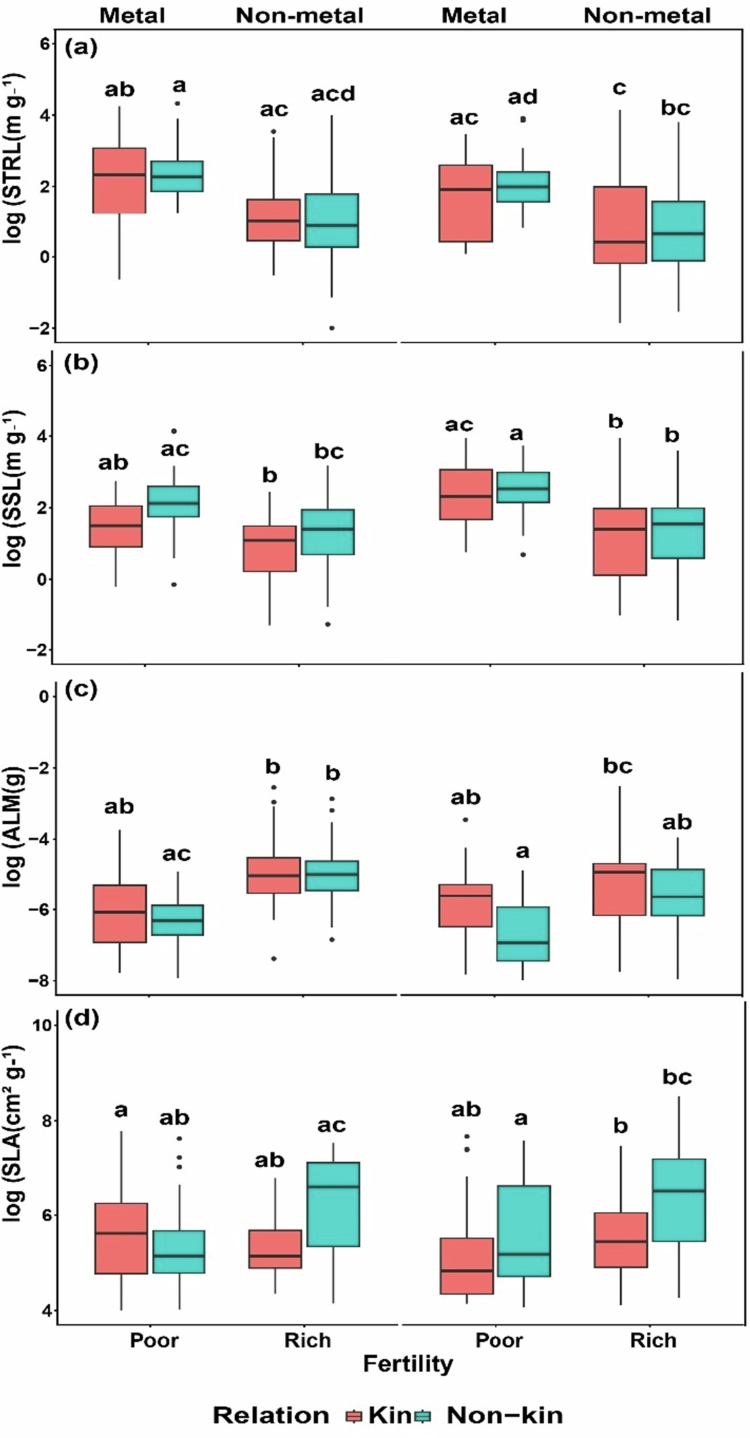
Resource acquisition efficiency parameters in all treatment combinations. (a) Specific taproot length (STRL), (b) specific stem length (SSL), (c) average leaf mass (ALM), and (d) specific leaf area(SLA) of kin and non-kin under poor versus rich soil fertility and metal treatments versus non-metal treatments. Common letters within each panel indicate no statistically significant difference among the treatment pairs in that panel (based on Tukey HSD tests on transformed data).

Specific taproot length (STRL) was affected by fertility and toxicity and was lower in more fertile soil (Table 2, Figure 3a). Kin plants had higher STRL than non-kin plants, and STRL was greater in poor soil compared to rich soil. Specific stem length (SSL) differed significantly according to kinship, fertility and toxicity (Table 2, Figure 3b): kin had lower SSL than non-kin, and plants grown in high-fertility soil had lower SSL than those in low-fertility soils. Toxicity had negative effect on SSL, and kin plant had lower SSL than non-kin plants under the toxicity treatments. ALM only responded to fertility being greater for kin combination pots, greater in more fertile soil and greater in more no-toxic soil, but the differences between kin and non-kin combinations were reduced under more fertile and under no-toxic soil (Table 2, Figure 3c). The specific leaf area (SLA) responded to kinship, fertility and toxicity, being higher in non-kin plants than in kin plants across fertility and toxicity treatments (Table 2, Figure 3d).

### 3.3 Photosynthetic traits

**Figure 4.**
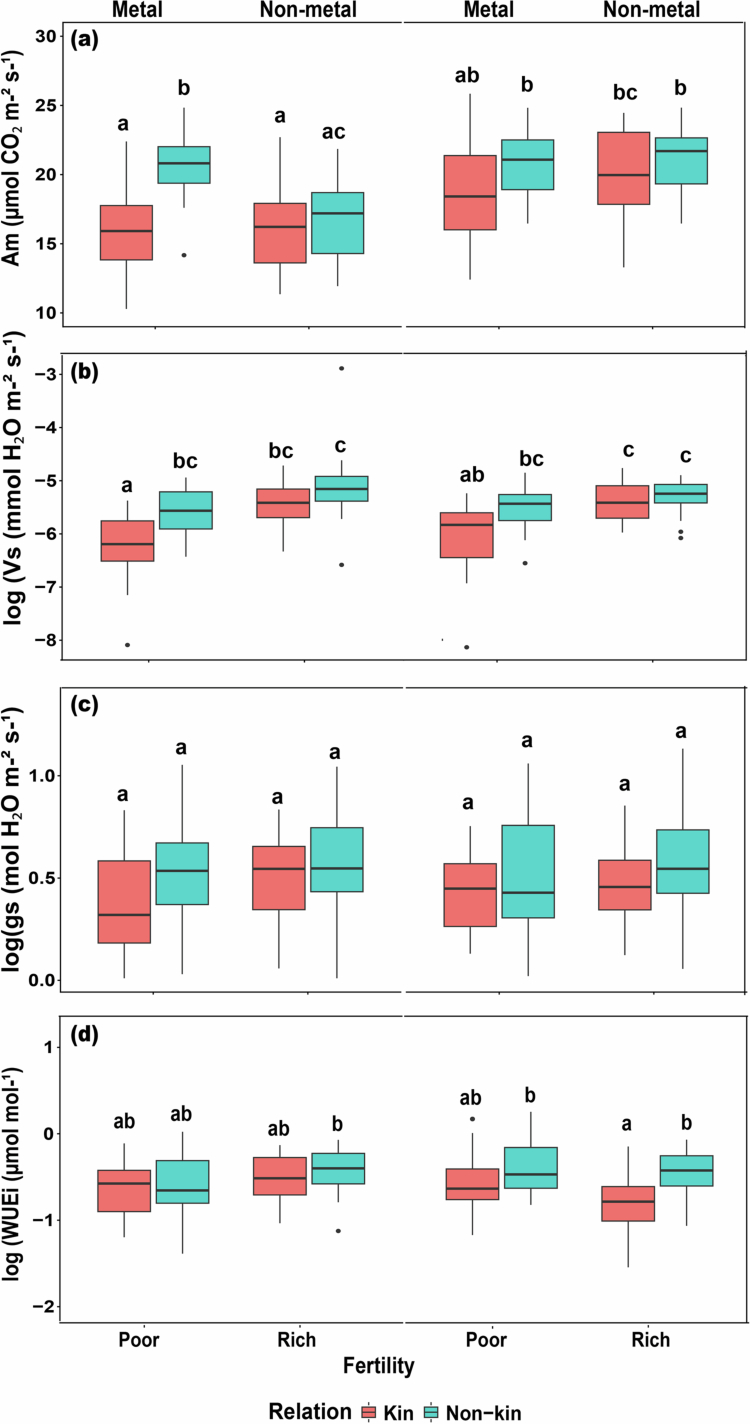
Photosynthetic parameters in all treatment combinations. (a) Assimilation rate (Am), (b) transpiration rate (Vs), (c) stomatal conductance (gs), and (d) WUEi (µmol mol⁻¹) of kin and non-kin under poor versus rich soil fertility and metal versus nonmetal treatments. Common letters within each panel indicate no statistically significant differences among the treatment pairs in that panel (based on Tukey HSD tests on transformed data).

The assimilation rate (*Am*) was affected by all treatments (kinship, fertility, and toxicity) and was higher in non-kin plants than in kin plants (Table 3, Figure 4a). Under toxic conditions, *Am* was significantly higher in non-kin plants, whereas no significant difference was observed between kin and non-kin plants in the no-toxicity treatment. Transpiration rate(*Vs*) was impacted by an interaction between kinship and fertility: *Vs* were significantly lower for kin than non-kin, was higher in more fertile soil, but the difference between kin and non-kin combinations decreased under more fertile conditions (Table 3, Figure 4b). Stomatal conductance (*gs*) was not significant across the treatments, but WUEi was significantly impacted by fertility and toxicity being higher for the non-kin plants than kin plants (Table 3, Figure 4c and d).

**Table 3.** Summarized statistical results for photosynthetic traits variables in the full model. Transformations applied to normalize response data prior to regression are indicated. Random effect standard deviances are given. Alternative case levels for fixed effects are indicated for coefficients. Coefficients are evaluated using t tests based on Satterthwaite approximation of error degrees of freedom.

	Am	Vs (log)	Gs	WUEi (log)
Random effects (variance)				
PotID	1.14	0.18	0.11	0.09
Genotype	0.70	0.14	0.12	0.03
Residual	2.73	0.45	0.20	0.16
Fixed effects				
Intercept	**15.922**	**−6.214**	**0.398**	**−0.622**
Relation: non-kin	**4.709**	**0.618**	0.121	0.021
Fertility: rich	0.416	**0.749**	0.103	0.147
Toxicity: non-metal	**2.502**	0.195	0.037	0.080
Relation: non-kin × fertility: rich	**−4.284**	−0.319	−0.090	0.090
Relation: non-kin × toxicity: non-metal	−2.232	−0.105	−0.082	0.122
Fertility: rich × toxicity: non-metal	1.268	−0.128	−0.020	−**0.346**
Relation: non-kin × fertility: rich × toxicity:non-metal	2.933	−0.108	0.125	0.164
R^2^m	0.40	0.33	0.04	0.15
R^2^c	0.45	0.48	0.43	0.40

Note: Bold values indicate coefficients significant at *p* < 0.05 are emboldened.

## 4 Discussion

### 4.1 Greater biomass accumulation for kin than non-kin plants

Our performance metrics (pot-level total biomass and shoot biomass) revealed strong treatment effects on kinship and fertility. When nutrient concentrations increased, biomass accumulation also increased,^11^^,^^13^ with differences being more pronounced in kin plants. Contrary to our initial expectation that non-kin plants would accumulate greater biomass due to reduced belowground competition,kin plants actually showed higher total and shoot biomass. This pattern is consistent with reduced competitive intensity among genetically related individuals, allowing kin plants to invest more in belowground traits (e.g. root mass fraction and specific taproot length) without sacrificing aboveground growth. These findings suggest that kin plants may optimize resource acquisition and allocation, translating efficient belowground investment into enhanced overall growth. These results are supported by the study of ^25,^ which showed that sibling plants exhibited enhanced growth performance in terms of biomass accumulation compared to non-sibling plants. This finding reinforces the idea that kin selection may increase plant fitness when grown with relatives.

The traditional understanding of plant resource allocation posits a trade-off between aboveground and belowground growth; increased investment in roots to compete for soil resources often comes at the expense of shoot development. For example, under drought, more biomass is allocated to roots, while under sufficient soil moisture, more is allocated to leaves, which is consistent with functional equilibrium theory.^50^ Root competition can lead to larger biomass reductions than can shoot competition, especially for smaller competitors.^51^ In our study, kin plants exhibited greater belowground investment (higher RMF and STRL), which might be expected to reduce aboveground biomass due to this trade-off. However, the observed increase in total and shoot biomass in kin groups suggests that this trade-off may not always apply or is mitigated by other factors associated with kin interactions.^12^ Any ‘cost’ of investing more into roots appears balanced or outweighed by benefits such as more efficient resource use or reduced energy expenditure on aggressive competition. This indicates that kin plants may adjust their root growth to exploit resources more efficiently and reduce unnecessary competition. Complementary resource uptake strategies or more efficient nutrient allocation within the plant may allow kin plants to achieve higher total biomass even with greater root investment.^1^^,^^52^ Therefore, the enhanced root traits observed in kin plants likely reflect an adaptive strategy for resource acquisition rather than harmful competition, ultimately supporting the greater overall growth and biomass accumulation observed in the kin groups.

### 4.2 Below-ground biomass allocation is greater for kin while above-ground allocation is greater for non-kin

Changes in biomass allocation can be difficult to interpret because increased allocation to roots may indicate stronger belowground competition for soil resources,^53^^,^^54^ yet it may also reflect reduced competitive inhibition if roots can freely explore the soil. By the same logic, increased allocation to leaf or stem can indicate increased competition for light,^55^ but it may also indicate increased performance, such that greater allocation to stem could lay the foundation for increased seed crops.^56^ In our study, lead toxicity significantly affected quinoa biomass allocation (Table 2). The most notable response was a reduction in RMF and STRL, where higher lead concentrations resulted in reduced investment in root biomass. This suppression occurred in both kin and non-kin combinations, indicating that lead toxicity disrupts normal belowground growth regardless of plant relatedness. Suppressed root allocation under toxic conditions may limit nutrient uptake, which could ultimately reduce future biomass or seed production. Importantly, despite these reductions, kin plants still allocated proportionally more biomass to roots (higher RMF and STRL), whereas non-kin plants allocated more biomass to stems and leaves (higher SMF and SSL). These findings suggest that kin plants maintained a belowground investment strategy to enhance nutrient access, while non-kin plants invested more in aboveground structures. The fact that toxicity reduced root allocation similarly in both kin and non-kin combinations suggests that soil contamination may interfere with belowground communication or root-to-root signaling.^57^ Elhakeem et al.^58^ found that plants growing with kin allocated more biomass to roots than non-self or unconnected plants did. An experiment on kin interactions of *Sorghum vulgare* grown under soil fertility and heavy metal stress found that kin plants had greater root allocation than non-kin in nutrient-poor soil.^13^ Both of these studies found results that agree with our own.

Quinoa naturally grows in dry, high-elevation environments where water and nutrient stress are major selective pressures, while light is abundant and not a limiting factor. Therefore, it is unlikely that quinoa has evolved phenotypic plasticity to respond to light competition.^10^^,^^59^^,^^60^ Instead, it is more likely to adjust its root system to cope with variation in belowground resources. In our study, increased allocation to stems in non-kin combinations may indicate a stronger investment in vertical growth, which is often associated with aboveground competition,^55^^,^^61^ but it could also reflect improved performance, as greater stem allocation can support higher potential seed production.^56^ Conversely, kin plants allocated more biomass to roots, potentially enhancing their ability to access soil nutrients. This pattern suggests that quinoa may suppress root growth less when growing with kin than with non-kin, pointing to a possible kin-facilitation or mutualistic effect.^10^

### 4.3 Resource acquisition traits were greater for kin plants while photosynthetic capacity was higher for non-kin plants

Resource acquisition traits are essential for plant survival because they determine how effectively plants capture water, nutrients, light, and other resources under different environmental conditions, including soil fertility gradients, moisture variation, and heavy metal stress.^62^ In our study, kin plants exhibited greater STRL than non-kin under nutrient-poor conditions, indicating enhanced belowground foraging ability. However, this difference was mainly evident in non-toxic soils. When soil toxicity increased, STRL was suppressed for both kin and non-kin, showing that lead contamination inhibits belowground performance and ultimately reduces plant fitness. Kin plants acquired more resources than non-kin under nutrient-poor soil, but this advantage diminished in nutrient-rich soil. This pattern is consistent with resource partitioning theory, which suggests that individuals sharing similar resource requirements – such as kin with similar genotypes, are more likely to compete strongly when resources are limited.^10^^,^^18^^,^^63^ Combined with our RMF results, this supports the idea that competition is stronger among kin because they use the same type of resources in similar ways (H1). A large body of literature indicates that plants elongate their roots more in low-nutrient soil to search the soil volume more efficiently for nutrients (e.g. ^3^^,^^31^^,^^39^^,^^64^^,^^65^). Root volume is directly related to the ability of plants to acquire soil nutrients.^66^^,^^67^ SSL was affected by all the treatments, whereas ALM was influenced only by kinship. Under both poor and rich nutrient conditions, both kin and non-kin plants showed higher SSL. Kin plants had slightly higher ALM when grown with kin compared to non-kin, but the difference was small. These patterns suggest that when resources are abundant, plants invest more in leaves to improve light capture and photosynthesis, an acquisitive strategy that supports faster growth.^50^^,^^68^

Surprisingly, photosynthetic parameters such as *As*, *Vs*, *gs*, VPD, WUEi, and SLA were higher for non-kin combinations relative to kin combinations, even though they had lower root mass allocation to support water uptake. This indicates that non-kin plants may invest more in aboveground traits that enhance photosynthetic efficiency and light capture. The higher SLA values observed in non-kin plants suggest thinner or larger leaves that facilitate greater light interception per unit mass, contributing to higher photosynthetic rates. However, despite this apparent physiological advantage, non-kin plants accumulated less biomass than kin plants, suggesting a trade-off between photosynthetic efficiency and biomass investment.

One possible explanation is that non-kin plants may allocate assimilated carbon toward maintaining competitive traits or stress-related metabolism rather than growth, consistent with the idea that higher SLA and photosynthetic capacity under competitive stress may reflect a resource-use strategy rather than enhanced productivity. In contrast, kin plants, which exhibited lower photosynthetic rates but higher biomass accumulation, may follow a more conservative allocation strategy that favors coordinated resource use and greater overall growth efficiency.^69^^,^^70^ Overall, our results show that although non-kin plants had higher photosynthetic rates and higher SLA, this did not lead to more growth or biomass. This suggests that having a higher photosynthetic capacity alone is not enough to ensure greater biomass production. In contrast, kin plants, despite having lower photosynthetic values, accumulated more biomass. This implies that growth may depend more on how efficiently plants use available resources rather than how much they capture through photosynthesis. Soil toxicity may also reduce the advantage of high SLA in non-kin plants, limiting their ability to convert photosynthetic activity into biomass. Future studies that examine both photosynthetic efficiency and how carbon is partitioned above- and belowground, along with signaling between roots, will be important for fully understanding these contrasting growth strategies.

## 5 Conclusion

We conclude that kin plants allocated more biomass to roots and also achieved higher total and shoot biomass, suggesting they were more effective at accessing soil resources. In contrast, non-kin plants had higher photosynthetic rates and greater specific leaf area (SLA), but this did not translate into increased growth. These results suggest that kin plants may prioritize belowground resource acquisition, supporting overall biomass accumulation, while non-kin plants invest more in leaf traits and photosynthetic efficiency, possibly as a competitive response, without necessarily increasing growth. Overall, our findings indicate that quinoa plants may achieve higher growth when grown with genetically similar neighbors under favorable conditions, although this effect is context-dependent. Further studies across diverse genotypes, environmental conditions, and field trials are needed to confirm these patterns and explore their potential implications for cultivation strategies.

## References

[1] Murphy GP, Dudley SA. Kin recognition: competition and cooperation in Impatiens (Balsaminaceae). Am J Bot. 2009;96:1990–1996. doi: 10.3732/ajb.0900006. 21622319

[2] Sher J, Zheng Y, Burns JH, Jan G, Zhang J-L. Kin recognition in plants-an ecological perspective: an overview of plant kin recognition under different resources, consequences and future challenges. J Plant Interact. 2025;20:2548579. doi: 10.1080/17429145.2025.2548579.

[3] Yang X, Li L, Xu Y, Kong C. Kin recognition in rice (Oryza sativa) lines. New Phytol. 2018;220:567–578. doi: 10.1111/nph.15296. 29956839

[4] Hamilton WD. The genetical evolution of social behaviour. II. J Theor Biol. 1964;7:17–52. doi: 10.1016/0022-5193(64)90039-6. 5875340

[5] Crepy MA, Casal JJ. Photoreceptor‐mediated kin recognition in plants. New Phytol. 2015;205:329–338. doi: 10.1111/nph.13040. 25264216

[6] Yamawo A, Mukai H. Outcome of interspecific competition depends on genotype of conspecific neighbours. Oecol. 2020;193:415–423. doi: 10.1007/s00442-020-04694-w. 32577823

[7] Casper BB, Jackson RB. Plant competition underground. Annu Rev Ecol Syst. 1997;28:545–570. doi: 10.1146/annurev.ecolsys.28.1.545.

[8] Depuydt S. Arguments for and against self and non-self root recognition in plants. Front Plant Sci. 2014;5:614. doi: 10.3389/fpls.2014.00614. 25414719 PMC4222137

[9] File AL, Murphy GP, Dudley SA. Fitness consequences of plants growing with siblings: reconciling kin selection, niche partitioning and competitive ability. Proc Biol Sci. 2012;279:209–218. doi: 10.1098/rspb.2011.1995. 22072602 PMC3223689

[10] Sher J, Khan N, Tomlinson KW. Plant growth of Chenopodium quinoa (Willd) is better when growing with kin than with non-kin regardless of soil nutrient conditions. Plant Ecol. 2024;225:153–161. doi: 10.1007/s11258-023-01386-2.

[11] Chen BJW, During HJ, Anten NPR. Detect thy neighbor: identity recognition at the root level in plants. Plant Sci. 2012;195:157–167. doi: 10.1016/j.plantsci.2012.07.006. 22921010

[12] Simonsen AK, Chow T, Stinchcombe JR. Reduced plant competition among kin can be explained by Jensen’s inequality. Ecol Evol. 2014;4:4454–4466. doi: 10.1002/ece3.1312. 25512842 PMC4264895

[13] Li J, Xu X, Feng R. Soil fertility and heavy metal pollution (Pb and Cd) alter kin interaction of Sorghum vulgare. Environ Exp Bot. 2018;155:368–377. doi: 10.1016/j.envexpbot.2018.05.009.

[14] Ehlers BK, Bilde T. Inclusive fitness, asymmetric competition and kin selection in plants. Oikos. 2019;128:765–774. doi: 10.1111/oik.06390.

[15] Willson MF, Hoppes WG, Goldman DA, Thomas PA, Katusic-Malmborg PL, Bothwell JL. Sibling competition in plants: an experimental study. Am Nat. 1987;129:304–311. doi: 10.1086/284636.

[16] Donohue K. The influence of neighbor relatedness on multilevel selection in the Great Lakes sea rocket. Am Nat. 2003;162:77–92. doi: 10.1086/375299. 12856238

[17] Dudley SA, File AL. Kin recognition in an annual plant. Biol Lett. 2007;3:435–438. doi: 10.1098/rsbl.2007.0232. 17567552 PMC2104794

[18] Sher J, Bibi F, Jan G, Tomlinson KW, Ayaz A, Zaman W. Kin and Non-kin connected plants benefit more than disconnected Kin and Non-kin plants under nutrient-competitive environments. Plants. 2023;12:487. doi: 10.3390/plants12030487. 36771572 PMC9920217

[19] Yamawo A, Sato M, Mukai H. Experimental evidence for benefit of self discrimination in roots of a clonal plant. AoB Plants. 2017;9:plx049. doi: 10.1093/aobpla/plx049.

[20] Allard RW, Adams J. Population studies in predominantly self-pollinating species. XIII. Intergenotypic competition and population structure in barley and wheat. Am Nat. 1969;103:621–645. doi: 10.1086/282630.

[21] Milla R, Forero DM, Escudero A, Iriondo JM. Growing with siblings: a common ground for cooperation or for fiercer competition among plants? Proc Biol Sci. 2009;276:2531–2540. doi: 10.1098/rspb.2009.0369. 19403541 PMC2686667

[22] Masclaux F, Hammond RL, Meunier J, Gouhier‐Darimont C, Keller L, Reymond P. Competitive ability not kinship affects growth of Arabidopsis thaliana accessions. New Phytol. 2010;185:322–331. doi: 10.1111/j.1469-8137.2009.03057.x. 19886895

[23] Argyres AZ, Schmitt J. Neighbor relatedness and competitive performance in Impatiens capensis (Balsaminaceae): a test of the resource partitioning hypothesis. Am J Bot. 1992;79:181–185. doi: 10.1002/j.1537-2197.1992.tb13636.x.

[24] McCall C, Mitchell‐Olds T, Waller DM. Fitness consequences of outcrossing in Impatiens capensis: tests of the frequency‐dependent and sib‐competition models. Evolution. 1989;43:1075–1084. 28564147 10.1111/j.1558-5646.1989.tb02552.x

[25] Cheplick GP, Kane KH. Genetic relatedness and competition in Triplasis purpurea (Poaceae): resource partitioning or kin selection? Int J Plant Sci. 2004;165:623–630. doi: 10.1086/386556.

[26] Palmer AG, Ali M, Yang S, Parchami N, Bento T, Mazzella A, Oni M, Riley MC, Schneider K, Massa N. Kin recognition is a nutrient-dependent inducible phenomenon. Plant Signal Behav. 2016;11:e1224045. doi: 10.1080/15592324.2016.1224045. 27552112 PMC5058466

[27] Müller I, Schmid B, Weiner J. The effect of nutrient availability on biomass allocation patterns in 27 species of herbaceous plants. Perspect Plant Ecol Evol Syst. 2000;3:115–127. doi: 10.1078/1433-8319-00007.

[28] Dybzinski R, Kelvakis A, McCabe J, Panock S, Anuchitlertchon K, Vasarhelyi L, McCormack ML, McNickle GG, Poorter H, Trinder C. How are nitrogen availability, fine‐root mass, and nitrogen uptake related empirically? Implications for models and theory. Global Change Biol. 2019;25:885–899. doi: 10.1111/gcb.14541. 30536492

[29] Rehling F, Sandner TM, Matthies D. Biomass partitioning in response to intraspecific competition depends on nutrients and species characteristics: a study of 43 plant species. J Ecol. 2021;109:2219–2233. doi: 10.1111/1365-2745.13635.

[30] O’Brien EE, Gersani M, Brown JS. Root proliferation and seed yield in response to spatial heterogeneity of below‐ground competition. New Phytol. 2005;168:401–412. doi: 10.1111/j.1469-8137.2005.01520.x. 16219079

[31] Murphy GP, Dudley SA. Above‐and below‐ground competition cues elicit independent responses. J Ecol. 2007;95:261–272. doi: 10.1111/j.1365-2745.2007.01217.x.

[32] Riaz M, Kamran M, Rizwan M, Ali S, Parveen A, Malik Z, Wang X. Cadmium uptake and translocation: synergetic roles of selenium and silicon in Cd detoxification for the production of low CD crops: a critical review. Chemosphere. 2021;273:129690. doi: 10.1016/j.chemosphere.2021.129690. 33524757

[33] Zhao H, Guan J, Liang Q, Zhang X, Hu H, Zhang J. Effects of cadmium stress on growth and physiological characteristics of sassafras seedlings. Sci Rep. 2021;11:1–11. doi: 10.1038/s41598-021-89322-0. 33972641 PMC8110755

[34] Singh S, Parihar P, Singh R, Singh VP, Prasad SM. Heavy metal tolerance in plants: role of transcriptomics, proteomics, metabolomics, and ionomics. Front Plant Sci. 2016;6:1143. doi: 10.3389/fpls.2015.01143. 26904030 PMC4744854

[35] Li S, Han X, Lu Z, Qiu W, Yu M, Li H, He Z, Zhuo R. MAPK Cascades and Transcriptional Factors: regulation of Heavy Metal Tolerance in Plants. Int J Mol Sci. 2022;23:4463. doi: 10.3390/ijms23084463. 35457281 PMC9032930

[36] Kapoor B, Kumar P, Gill NS, Sharma R, Thakur N, Irfan M. Molecular mechanisms underpinning the silicon-selenium (Si-Se) interactome and cross-talk in stress-induced plant responses. Plant Soil. 2023;486:45–68. doi: 10.1007/s11104-022-05482-6.

[37] Hinojosa L, González JA, Barrios-Masias FH, Fuentes F, Murphy KM. Quinoa abiotic stress responses: a review. Plants. 2018;7:106. doi: 10.3390/plants7040106. 30501077 PMC6313892

[38] Gámez AL, Soba D, Zamarreño ÁM, García-Mina JM, Aranjuelo I, Morales F. Effect of water stress during grain filling on yield, quality and physiological traits of Illpa and Rainbow quinoa (Chenopodium quinoa Willd.) cultivars. Plants. 2019;8:173. doi: 10.3390/plants8060173. 31207888 PMC6631622

[39] Cai Z-Q, Poorter L, Cao K-F, Bongers F. Seedling growth strategies in Bauhinia species: comparing lianas and trees. Ann Bot. 2007;100:831–838. doi: 10.1093/aob/mcm179. 17720978 PMC2749636

[40] Parwada C, Mandumbu R, Tibugari H, Badze D, Mhungu S. Effect of soil fertility amendment, planting density and growing season on Chenopodium quinoa Willd (Quinoa) in Zimbabwe. Cogent Food Agric. 2020;6:1792668. doi: 10.1080/23311932.2020.1792668.

[41] Wali AM, Kenawey MK, Ibrahim OM, Abd El Lateef EM. Productivity of Quinoa (Chenopodium quinoa L.) under new reclaimed soil conditions at north-western coast of Egypt. Bull Natl Res Cent. 2022;46:1–8. doi: 10.1186/s42269-022-00724-0.

[42] Abbas G, Amjad M, Saqib M, Murtaza B, Asif Naeem M, Shabbir A, Murtaza G. Soil sodicity is more detrimental than salinity for quinoa (Chenopodium quinoa Willd.): a multivariate comparison of physiological, biochemical and nutritional quality attributes. J Agron Crop Sci. 2021;207:59–73. doi: 10.1111/jac.12451.

[43] Abdal N, Abbas G, Asad SA, Ghfar AA, Shah GM, Rizwan M, Ali S, Shahbaz M. Salinity mitigates cadmium-induced phytotoxicity in quinoa (Chenopodium quinoa Willd.) by limiting the Cd uptake and improved responses to oxidative stress: implications for phytoremediation. Environ Geochem Health. 2021;45:1–15. doi: 10.1007/s10653-021-01082-y. 34476635

[44] Cai Z-Q, Gao Q. Comparative physiological and biochemical mechanisms of salt tolerance in five contrasting highland quinoa cultivars. BMC Plant Biol. 2020;20:1–15. 32050903 10.1186/s12870-020-2279-8 PMC7017487

[45] Tingting X, Peixi SU, Lishan S. Photosynthetic characteristics and water use efficiency of sweet sorghum under different watering regimes. Pak J Bot. 2010;42:3981–3994.

[46] Du T, Meng P, Huang J, Peng S, Xiong D. Fast photosynthesis measurements for phenotyping photosynthetic capacity of rice. Plant Methods. 2020;16:6. doi: 10.1186/s13007-020-0553-2. 31998402 PMC6979334

[47] Nakagawa S, Schielzeth H. A general and simple method for obtaining R2 from generalized linear mixed‐effects models. Methods Ecol Evol. 2013;4:133–142. doi: 10.1111/j.2041-210x.2012.00261.x.

[48] Nakagawa S, Johnson PCD, Schielzeth H. The coefficient of determination R 2 and intra-class correlation coefficient from generalized linear mixed-effects models revisited and expanded. J R Soc Interface. 2017;14:20170213. doi: 10.1098/rsif.2017.0213. 28904005 PMC5636267

[49] Piepho H-P. An algorithm for a letter-based representation of all-pairwise comparisons. J Comput Graph Stat. 2004;13:456–466. doi: 10.1198/1061860043515.

[50] Asefa M, Worthy SJ, Cao M, Song X, Lozano YM, Yang J. Above-and below-ground plant traits are not consistent in response to drought and competition treatments. Ann Bot. 2022;130:939–950. doi: 10.1093/aob/mcac108. 36001733 PMC9851322

[51] Kiær LP, Weisbach AN, Weiner J. Root and shoot competition: a meta‐analysis. J Ecol. 2013;101:1298–1312. doi: 10.1111/1365-2745.12129.

[52] Freschet GT, Swart EM, Cornelissen JHC. Integrated plant phenotypic responses to contrasting above‐and below‐ground resources: key roles of specific leaf area and root mass fraction. New Phytol. 2015;206:1247–1260. doi: 10.1111/nph.13352. 25783781

[53] Rajaniemi TK. Why does fertilization reduce plant species diversity? Testing three competition‐based hypotheses. J Ecol. 2002;90:316–324. doi: 10.1046/j.1365-2745.2001.00662.x.

[54] Qi Y, Wei W, Chen C, Chen L. Plant root-shoot biomass allocation over diverse biomes: a global synthesis. Glob Ecol Conserv. 2019;18:e00606. doi: 10.1016/j.gecco.2019.e00606.

[55] De Vries J, Evers JB, Poelman EH, Anten NPR. Optimal plant defence under competition for light and nutrients: an evolutionary modelling approach. In Silico Plants. 2020;2:diaa008. doi: 10.1093/insilicoplants/diaa008.

[56] Preece C, Livarda A, Christin P, Wallace M, Martin G, Charles M, Jones G, Rees M, Osborne CP. How did the domestication of Fertile Crescent grain crops increase their yields? Funct Ecol. 2017;31:387–397. doi: 10.1111/1365-2435.12760. 28286354 PMC5324541

[57] Panda SK, Baluška F, Matsumoto H. Aluminum stress signaling in plants. Plant Signal Behav. 2009;4(7):592–597. doi: 10.4161/psb.4.7.8903. 19820334 PMC2710549

[58] Elhakeem A, Markovic D, Broberg A, Anten NPR, Ninkovic V. Aboveground mechanical stimuli affect belowground plant-plant communication. PLoS One. 2018;13:e0195646. doi: 10.1371/journal.pone.0195646. 29718944 PMC5931455

[59] Kurashige NS, Agrawal AA. Phenotypic plasticity to light competition and herbivory in Chenopodium album (Chenopodiaceae). Am J Bot. 2005;92:21–26. doi: 10.3732/ajb.92.1.21. 21652380

[60] Bongers FJ, Douma JC, Iwasa Y, Pierik R, Evers JB, Anten NPR. Variation in plastic responses to light results from selection in different competitive environments—a game theoretical approach using virtual plants. PLoS Comput Biol. 2019;15:e1007253. doi: 10.1371/journal.pcbi.1007253. 31433817 PMC6703680

[61] Bebre I, Marques I, Annighöfer P. Biomass allocation and leaf morphology of saplings grown under various conditions of light availability and competition types. Plants. 2022;11:305. doi: 10.3390/plants11030305. 35161289 PMC8839049

[62] Zhang Z, Liu Y, Hardrath A, Jin H, Van Kleunen M. Increases in multiple resources promote competitive ability of naturalized non-native plants. Commun Biol. 2022;5:1150. doi: 10.1038/s42003-022-04113-1. 36310319 PMC9618556

[63] Young Z, Jpw Y. Sib competition can favour sex in two ways. J Theoret Biol. 1981;88:755–756. 7266014 10.1016/0022-5193(81)90249-6

[64] Robinson D, Hodge A, Griffiths BS, Fitter AH. Plant root proliferation in nitrogen–rich patches confers competitive advantage. Proc R Soc Lond B Biol Sci. 1999;266:431–435. doi: 10.1098/rspb.1999.0656.

[65] File AL, Klironomos J, Maherali H, Dudley SA. Plant kin recognition enhances abundance of symbiotic microbial partner. PLoS ONE. 2012;7(9). doi: 10.1371/journal.pone.0045648. PMC3460938 23029158

[66] Craine JM, Elmore AJ, Aidar MPM, Bustamante M, Dawson TE, Hobbie EA, Kahmen A, Mack MC, McLauchlan KK, Michelsen A. Global patterns of foliar nitrogen isotopes and their relationships with climate, mycorrhizal fungi, foliar nutrient concentrations, and nitrogen availability. New Phytol. 2009;183:980–992. doi: 10.1111/j.1469-8137.2009.02917.x. 19563444

[67] Milla R, del Burgo AV, Escudero A, Iriondo JM. Kinship rivalry does not trigger specific allocation strategies in Lupinus angustifolius. Ann Bot. 2012;110:165–175. doi: 10.1093/aob/mcs093. 22562807 PMC3380590

[68] Wright IJ, Reich PB, Westoby M, Ackerly DD, Baruch Z, Bongers F, Cavender-Bares J, Chapin T, Cornelissen JHC, Diemer M. The worldwide leaf economics spectrum. Nature. 2004;428:821–827. doi: 10.1038/nature02403. 15103368

[69] Thompson RA. A neutral theory of plant carbon allocation. Tree Physiol. 2024;44:tpad151. doi: 10.1093/treephys/tpad151. 38102767

[70] Avalos G, Frazer K, Le Gall H. Plant size influences specific leaf area in palms: a case for the diminishing returns hypothesis. Oecologia. 2025;207:56. doi: 10.1007/s00442-025-05698-0. 40153038

